# Second-line glucose-lowering drugs added to metformin and the risk of hospitalization for heart failure: A nationwide cohort study

**DOI:** 10.1371/journal.pone.0211959

**Published:** 2019-02-11

**Authors:** Su Jin Lee, Kyoung Hwa Ha, Jung Hyun Lee, Hokyou Lee, Dae Jung Kim, Hyeon Chang Kim

**Affiliations:** 1 Department of Internal Medicine, Yonsei University College of Medicine, Seoul, Korea; 2 Department of Preventive Medicine, Yonsei University College of Medicine, Seoul, Korea; 3 Department of Endocrinology and Metabolism, Ajou University School of Medicine, Suwon, Korea; 4 Cardiovascular and Metabolic Disease Etiology Research Center, Ajou University School of Medicine, Suwon, Korea; 5 Cardiovascular and Metabolic Disease Etiology Research Center, Yonsei University College of Medicine, Seoul, Korea; International University of Health and Welfare, School of Medicine, JAPAN

## Abstract

**Aim:**

To compare the risks of hospitalization for heart failure (HHF) associated with sulfonylurea (SU), dipeptidyl peptidase-4 inhibitor (DPP-4i), and thiazolidinedione (TZD) as add-on medications to metformin (MET) therapy using the data of Korean adults with type-2 diabetes from the Korean National Health Insurance database.

**Methods:**

We identified 98,383 people who received SU (n = 42,683), DPP-4i (n = 50,310), or TZD (n = 5,390) added to initial treatment of MET monotherapy in patients with type-2 diabetes. The main outcome was the hospitalization for HHF. Hazard ratios for HHF by type of second-line glucose-lowering medication were estimated by Cox-proportional hazard models. Sex, age, duration of MET monotherapy, Charlson Comorbidity Index and additional comorbidities, and calendar year were controlled as potential confounders.

**Results:**

The observed numbers (rate per 100,000 person-years) of HHF events were 1,129 (658) for MET+SU users, 710 (455) for MET+DPP-4i users, and 110 (570) for MET+TZD users. Compared to that for MET+SU users (reference group), the adjusted hazard ratios for HHF events were 0.76 (95% confidence interval 0.69–0.84) for MET+DPP-4i users and 0.96 (95% confidence interval 0.79–1.17) for MET+TZD users.

**Conclusion:**

DPP-4i as an add-on therapy to MET may lower the risks of HHF compared with SU.

## Introduction

Heart failure (HF) is one of the main health burdens of cardiovascular disease (CVD) in people with type-2 diabetes mellitus (T2D) [1]. Pathogenic elements central to HF in T2D are hypertension, atherosclerotic vascular disease, and diabetic cardiomyopathy related to insulin resistance. Although HF may be one of several fatal complications of T2D, HF could be preventable or treatable with drugs [2]. Thus, identifying the appropriate drug for glycemic control is important in the treatment of T2D to prevent HF.

Metformin (MET) is the most commonly used starting drug for patients with T2D on the basis of international guidelines [3]. In Korea, MET is the preferred initial oral antidiabetic drug, and it has also been the most commonly prescribed antidiabetic medication since 2010 [4, 5]. In addition, in 2016, >80% of dual therapy regimens included MET as the first-line therapy. As second-line therapies added to MET, sulfonylurea (SU), dipeptidyl peptidase-4 inhibitor (DPP-4i), and thiazolidinedione (TZD) have been most frequently prescribed in Korea according to data from the Korean Diabetes Association [4]. MET+SU was the most common dual therapy protocol until 2014. However, at present, MET+DPP-4i is the most frequently prescribed dual therapy.

DPP-4i is commonly used because it is associated with a relatively low risk of hypoglycemia and weight gain [6]. However, there are controversies regarding the HF risk associated with DPP-4i. The Saxagliptin Assessment of Vascular Outcomes Recorded in Patients with Diabetes Mellitus–Thrombolysis in Myocardial infarction 53 (SAVOR-TIMI 53) trial demonstrated that saxagliptin was associated with increased rates of hospitalization for HF (HHF) [7]. The Examination of CV Outcomes with Alogliptin versus Standard of Care (EXAMINE) trial [8] and the Trial Evaluating CV Outcomes with Sitagliptin trial revealed that alogliptin and sitagliptin did not increase the risk of HHF [9]. These discrepancies could have been caused by the different study designs and populations examined.

Our previous study revealed that MET+DPP-4i for T2D was associated with a lower CVD risk than that of MET+SU [10]. HF is a frequent and serious comorbidity of T2D that can be fatal. Considering this, we investigated the effect of DPP-4i and TZD as add-on therapies to MET in T2D patients on the risk of HHF using real-world data.

## Materials and methods

This study was approved by the Institutional Review Board (IRB) of the Yonsei University Health System (IRB number 4-2015-1023). All datasets were anonymous and de-identified. As such, the need for informed consent was waived.

### Data and study population

The National Health Insurance Service (NHIS) covers approximately 50 million people in Korea. In the NHIS claims database, diagnoses are coded using the International Classification of Disease version 10 (ICD-10). Insured individuals are required to undergo general health screenings every 2 years [11]. The health screening dataset includes information on height, weight, waist circumference, blood pressure, fasting glucose level, cholesterol level, health behaviors, and personal history of disease.

**Fig 1:**
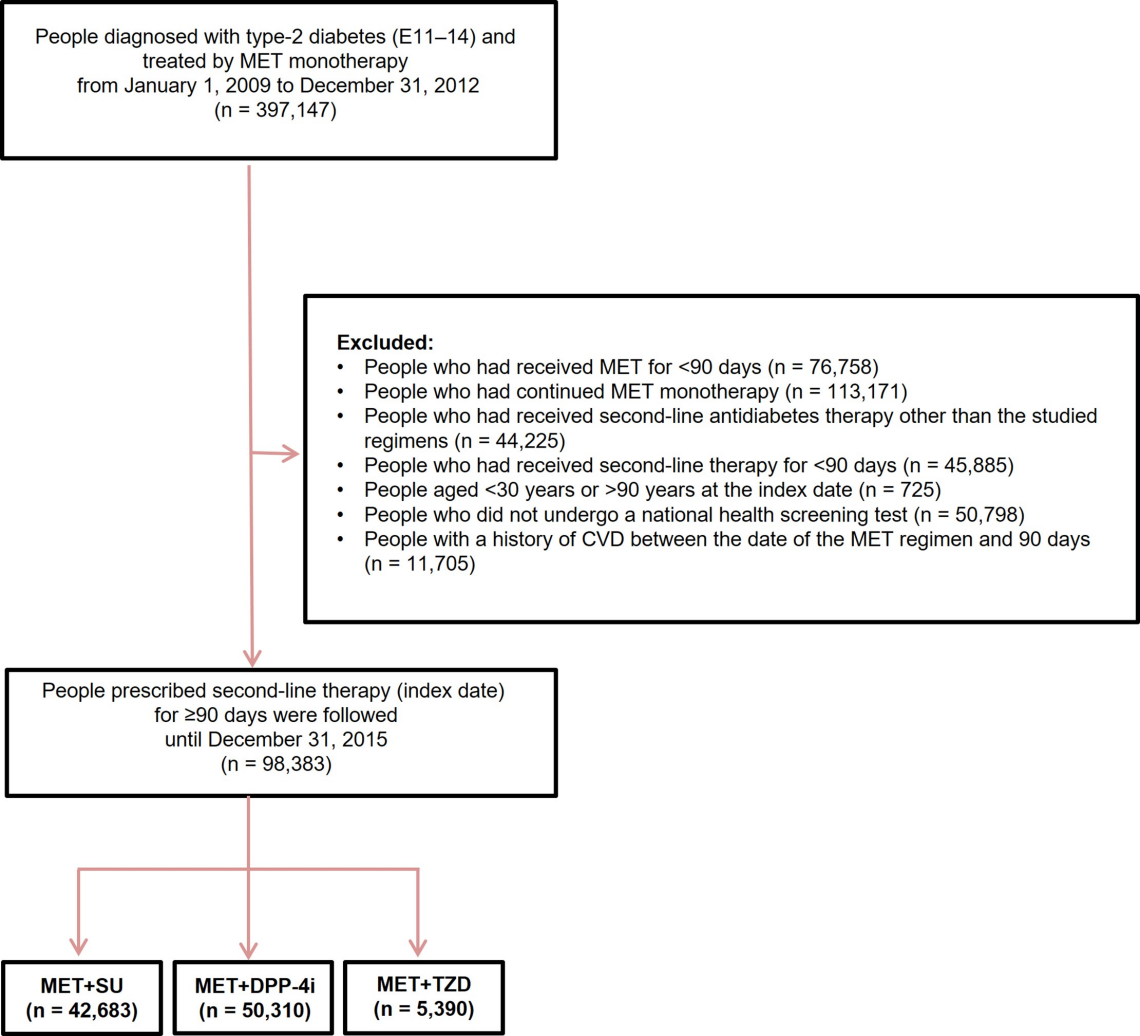
Diagram of study flow. MET, metformin; CVD, cardiovascular disease; SU, sulfonylurea; DPP-4i, dipeptidyl peptidase-4 inhibitor; TZD, thiazolidinedione.

In Korea, MET is the most commonly prescribed drug in Korea [5]. MET can be prescribed as a first-line glucose-lowering treatment for T2D under health insurance coverage when the patient’s glycated hemoglobin is <53 mmol/mol (7.0%). Dual therapy can be started only if the glycemic target is not achieved despite MET monotherapy for at least 2–4 months. We selected T2D (ICD-10 codes E11–E14) and included patients treated with MET monotherapy for ≥90 days between January 2009 and December 2012 (n = 397,147) (Fig 1). The main exposure was second-line glucose-lowering drugs added to MET for ≥90 days, and the initiation date of second-line glucose-lowering drugs after MET monotherapy for 90 days was defined as the index date. We selected individuals aged 30–90 years at the index date. Second-line glucose-lowering drugs of interest were limited to the three most common drugs: SU, DPP-4i, and TZD. We excluded individuals who continued MET monotherapy without second-line add-on. Then, people prescribed second-line glucose-lowering therapies, including insulin and drugs other than SU, DPP-4i, and TZD were excluded. We also excluded people with the event of CVD within 90 days after the index date. Three groups were followed until the end of study, disregarding any regimen changes over time (intention-to-treat). Also, we used “on-treatment” analysis, which follow-up is censored at the time when the drug is changed [12]. The study outcome was the first occurrence of HHF (ICD-10: I50). The follow-up time was defined as the period between the index date and the study outcome. The end of the study period was 31 December 2015. Patients who died before the end of study date were censored. Glucose-lowering drugs were identified by the relevant Anatomic Therapeutic Chemical (ATC) code, a classification system for drugs created by the World Health Organization Collaborating Center for Drug Statistics Methodology. The following ATC codes were used in this study: A10BA02 for MET; A10BB and A10BD02 for SU; A10BH, A10BD07, A10BD08, A10BD10, A10BD11, A10BD13, and A10BD18 for DPP-4i; and A10BG, A10BD03, and A10BD05 for TZD.

### Covariates

Comorbidities were adjusted using the Charlson Comorbidity Index and calculated by individual conditions, such as hypertension (ICD-10 codes I10–I15 and/or ATC codes C02–C03 and C07–C09), dyslipidemia (ICD-10 code E78 and/or ATC code C10), atrial fibrillation (ICD-10 code I48), chronic kidney disease (ICD-10 code N18), and microvascular complications of diabetes, including diabetic retinopathy (ICD-10 codes E10.3, E11.3, E12.3, E13.3, E14.3, and H36.0), diabetic neuropathy (ICD-10 codes E10.4, E11.4, E12.4, E13.4, E14.4, and G63.2), and diabetic nephropathy (ICD-10 codes E10.2, E11.2, E12.2, E13.2, E14.2, and N08.3). CVD included myocardial infarction (ICD-10 codes I21–I23), cerebrovascular diseases (ICD-10 codes E10.4, E11.4, E12.4, E13.4, E14.4, and G63.2), unstable angina (ICD-10 code I20.0), HF (ICD-10 code I50), and transient cerebral ischemic attack (ICD-10 code G45).

A subgroup analysis was performed to account for additional covariates, such as body mass index (BMI), waist circumference, systolic blood pressure, total cholesterol level, high-density lipoprotein (HDL) cholesterol level, low-density lipoprotein (LDL) cholesterol level, triglyceride level, fasting glucose level, serum creatinine level, smoking status (non-smoker, former smoker, or current smoker), and family history of stroke and heart disease. These variables were measured via health screening within 1 year before the index date. We used available data from patients who had undergone health screening (n = 35,297).

### Statistical analyses

Baseline characteristics were compared between patients treated with the different second-line glucose-lowering drugs. Cox proportional hazard regression models were used to calculate the hazard ratios (HRs) and 95% confidence intervals (CIs) for the incidence of HHF. We tested the proportional hazards assumption on the basis of Schoenfeld residuals [13]. The MET+SU regimen was used as a reference group for comparisons with the other second-line regimens. Potential confounders were sequentially adjusted in two statistical models, as follows: model 1 = sex and age; model 2 = sex, age, duration of MET therapy, hypertension, dyslipidemia, atrial fibrillation, chronic kidney disease, microvascular complications of diabetes (retinopathy, neuropathy, and nephropathy), previous history of CVD, Charlson Comorbidity Index, and calendar index year. All analyses were conducted using SAS software, version 9.4 (SAS Institute Inc., Cary, NC, USA).

## Results

### Risk of hospitalization for heart failure by claim data

The patients were divided into the following three groups: MET+SU (n = 42,683), MET+DPP-4i (n = 50,310), and MET+TZD (n = 5,390). The mean index age of the study population was 58.3 ± 11.1 years, and 53.4% were men. Compared to MET+SU users, MET+DPP-4i users and MET+TZD users were younger, had a higher prevalence of microvascular complications such as diabetic retinopathy, neuropathy, and nephropathy, had a lower prevalence of hypertension, and had a higher prevalence of dyslipidemia (Table 1). Over 346,991 person-years of follow-up, 1,949 HHF events were observed. The sex and age-adjusted HR of experiencing an HHF event was 0.80 (95% CI 0.73–0.88) for MET+DPP-4i therapy compared to MET+SU therapy (reference). This lower HR risk remained statistically significant after additional adjustments. The risk of HHF in MET+TZD users (sex and age-adjusted HR 0.94, 95% CI 0.78–1.15) was lower than that in MET+SU users without statistical significance (Table 2). By using “on-treatment” analysis as mentioned in method, the use of MET+DPP-4i compared to MET+SU was associated with a 16% lower rate of development of heart failure consistent to the intention-to-treat that we used (S1 Table). Cumulative risk of HHF was significantly lower in MET+DPP-4i compared with MET+SU (S1 Fig).

**Table 1:** Baseline characteristics by type of second-line antidiabetic medication.

	Total (n = 98,383)	MET+SU (n = 42,683)	MET+DPP4i (n = 50,310)	MET+TZD (n = 5,390)
**Men**	52,520 (53.4)	22, 581 (52.9)	26,946 (53.6)	2,993 (55.5)
**Age, years**	58.3 ± 11.1	59.6 ± 11.3	57.2 ± 10.9	57.6 ± 11.1
**Inclusion year**				
2009	49,960 (50.8)	21,827 (51.1)	25,030 (49.8)	3,103 (57.6)
2010	26,278 (26.7)	11,025 (25.8)	13,953 (27.7)	1,300 (24.1)
2011	13,271 (13.5)	5,679 (13.3)	7,000 (13.9)	592 (11.0)
2012	8,874 (9.0)	4,152 (9.7)	4,327 (8.6)	395(7.3)
**Comedications**				
Statin therapy	58,357 (59.3)	22,681 (53.1)	32,229 (64.1)	3,447 (64.0)
ACE inhibitors	5,334 (5.4)	2,683 (6.3)	2,362 (4.7)	289 (5.4)
ARB	54,986 (55.9)	23,978 (56.2)	28,017 (55.7)	2,991 (55.5)
Loop diuretics	5,883 (6.0)	2,986 (7.0)	2,560 (5.1)	337 (6.3)
Beta blockers	20,610 (20.9)	9,568 (22.4)	9,963 (19.8)	1,079 (20.0)
Dihydropyridines	29,692 (30.2)	15,427 (36.1)	12,689 (25.2)	1,576 (29.2)
Non-hydropyridines	3,173 (3.2)	1,343 (3.2)	1,676 (3.3)	154 (2.9)
Low dose acetylic salicylic acid	39,745 (40.4)	18,001 (42.2)	19,659 (39.1)	2,085 (38.7)
Warfarin	1,268 (1.3)	573 (1.3)	643 (1.3)	52 (1.0)
**Comorbidities**				
Hypertension	69,470 (70.6)	30,811 (72.2)	34,906 (69.4)	3,753 (69.6)
Dyslipidemia	70,418 (71.6)	27,489 (64.4)	38,911 (77.3)	4,018(74.6)
Atrial fibrillation	1,780 (1.8)	724 (1.7)	991 (2.0)	102 (1.9)
Chronic kidney disease	779 (0.8)	295 (0.7)	419 (0.8)	65 (1.2)
Diabetic retinopathy	11,416 (11.6)	4,505 (10.6)	6,301 (12.5)	610 (11.3)
Diabetic neuropathy	8,251 (8.4)	3,449 (8.1)	4,336 (8.6)	466 (8.7)
Diabetic nephropathy	4,550 (4.6)	1,313 (3.1)	2,949 (5.9)	288 (5.3)
Cardiovascular disease	16,184 (16.4)	7,227 (16.9)	8,065 (16.0)	892 (16.6)
**Charlson score, unit**	2.9 ± 1.6	2.8 ± 1.6	2.9 ± 1.6	3.0 ± 1.6

Values are presented as the mean ± standard deviation or n (%). MET, metformin; SU, sulfonylurea; DPP-4i, dipeptidyl peptidase-4 inhibitor; TZD, thiazolidinedione; ACE inhibitor, angiotensin-converting enzyme inhibitor; ARB, aldosterone receptor blocker.

**Table 2:** Hazard ratios for the development of heart failure by type of second-line antidiabetic medication.

Drugs	Person-years	No. of cases	Event rate (per 100,000 PY)	Adjusted 1*	Adjusted 2†
				HR (95% CI)	HR (95% CI)
MET+SU	171516	1129	658	1.00	1.00
MET+DPP-4i	156163	710	455	0.80 (0.73–0.88)	0.76 (0.69–0.84)
MET+TZD	19312	110	570	0.94 (0.78–1.15)	0.96 (0.79–1.17)

MET, metformin; SU, sulfonylurea; DPP-4i, dipeptidyl peptidase-4 inhibitor; TZD, thiazolidinedione; HR, hazard ratio; PY, person-year; CI, confidence interval; no., number.

*Adjusted for sex and age.

†Adjusted for sex, age, the duration of metformin therapy, hypertension, dyslipidemia, atrial fibrillation, chronic kidney disease, microvascular complications of diabetes (retinopathy, neuropathy, or nephropathy), cardiovascular disease, the Charlson Comorbidity Index, and calendar index year.

### Analysis of the risk of HHF stratified by history of CVD

To assess whether a history of CVD affected the relationship between the types of add-on medication and the risk of HHF, we repeated the analysis separately for people with and those without a history of CVD (Table 3). Compared with use of MET+SU, the use of MET+DPP-4i was associated with an 18% lower rate of HHF in the group with a history of CVD (HR 0.82, 95% CI 0.70–0.96) and a 28% lower rate of HHF in the group without a history of CVD (HR 0.72; 95% CI 0.63–0.82). The risk of HHF in MET+TZD users compared to MET+SU was higher in group with CVD history, and lower in group without CVD history, without significance.

**Table 3:** Hazard ratios for the development of heart failure by type of second-line antidiabetic medication according to history of cardiovascular disease.

Drugs	CVD history (n = 16,184)				No CVD history (n = 82,199)			
	Person-years	No. of cases	Event rate (per 100,000 PY)	HR (95% CI)	Person-years	No. of cases	Event rate (per 100,000 PY)	HR (95% CI)
MET+SU	27,936	415	1,486	1.00	143,580	714	497	1.00
MET+DPP-4i	24,100	292	1,212	0.82 (0.70–0.96)	132,063	418	317	0.72 (0.63–0.82)
MET+TZD	2,914	45	1,544	1.10 (0.81–1.50)	16,398	65	396	0.88 (0.68–1.14)

CVD, cardiovascular disease; MET, metformin; SU, sulfonylurea; DPP-4i, dipeptidyl peptidase-4 inhibitor; TZD, thiazolidinedione; HR, hazard ratio; CI, confidence interval; PY, person-year; no., number. Data are adjusted for sex, age, the duration of MET therapy, hypertension, dyslipidemia, atrial fibrillation, chronic kidney disease, microvascular complications of diabetes (retinopathy, neuropathy, or nephropathy), cardiovascular disease, the Charlson Comorbidity Index, and calendar index year.

### Subgroup analysis using national health screening data

The NHIS claims data included all physician-diagnosed disorders coded by ICD-10 but did not include objective data on exposure levels to CV risk factors. Therefore, we performed a subgroup analysis with available health screening data. In total, 35,297 people (35.9%) remained with available screening data and 498 per 100,000 person-years experienced HHF. Using MET+SU as a reference group, the HRs for HHF adjusted by age and sex were 0.74 (95% CI 0.61–0.89) for MET+DPP-4i and 1.11 (95% CI 0.77–1.59) for MET+TZD, respectively (Table 4). Moreover, the lowered HHF risks of the MET+DPP-4i group were also observed in comparison to the MET+SU group after adjusting for additional covariates. The baseline characteristics of patients treated with the different second-line drugs are shown in S2 Table.

**Table 4:** Hazard ratios for the development of heart failure by type of second-line antidiabetic medication in patients with available health screening data.

Drugs	Person-years	No. of cases	Event rate (per 100,000 PY)	Adjusted 1*	Adjusted 2†
				HR (95% CI)	HR (95% CI)
MET+SU	57,209	280	489	1.00	1.00
MET+DPP-4i	59,428	185	311	0.74 (0.61–0.89)	0.65 (0.54–0.79)
MET+TZD	6,949	33	475	1.11 (0.77–1.59)	1.05 (0.73–1.51)

MET, metformin; SU, sulfonylurea; DPP-4i, dipeptidyl peptidase-4 inhibitor; TZD, thiazolidinedione; HR, hazard ratio; CI, confidence interval; PY, person-year; no., number

*Adjusted for sex and age.

†Adjusted for sex, age, the duration of metformin therapy, body mass index, waist circumference, systolic blood pressure, total cholesterol level, high-density lipoprotein cholesterol level, low-density lipoprotein cholesterol level, triglyceride level, fasting glucose level, serum creatinine level, smoking status, family history of stroke and heart disease, history of cardiovascular disease, Charlson Comorbidity Index, and calendar index year.

## Discussion

The present study of nationwide real-world data demonstrated that people with T2D who were prescribed DPP-4i as a second-line drug alongside MET therapy had relatively lower risks of HHF than did those who were prescribed SU regardless of their CV history. These findings were directionally consistent across the subgroups that were available for further analysis using health screening data. Additionally, people who were prescribed TZD tended to have lower risks without statistical significance. This result could be explained by the fact that TZD was not prescribed to people at high risk of HHF because of doctors’ concerns about the high risks of HHF associated with TZD reported in previous studies [14, 15].

Many previous studies also reported the risk of HHF during monotherapy with glucose-lowering drugs [16–18]. Fu *et al*. revealed that individuals treated with DPP-4i had significantly lower risks of HHF among patients with no baseline CVD. The risk of HHF was lower in those treated with DPP-4i monotherapy than SU, pioglitazone, or insulin therapy alone [19]. When comparing the outcomes of second-line medication added to MET, MET+DPP-4i yielded a lower HR than MET+SU in terms of all-cause mortality, stroke, cancer, and the combined endpoint [10, 20, 21]. Recently, using Korean NHIS claim data, treatment with DPP-4i has been shown to be associated with a lower HF risk than treatment with SU [22]. Our previous study also demonstrated CVD risk reduction associated with MET+DPP-4i treatment for diabetes when compared to MET+SU [10].

As the NHIS health care utilization database did not include laboratory or anthropometric parameters, we employed strategies to maximize comparability between the three study groups. First, we limited our analysis to people treated using only one second-line therapy in addition to MET. Second, we excluded people who were not prescribed MET monotherapy before combination therapy or those who used MET monotherapy for <90 days. Third, we adjusted for the duration of MET monotherapy as a potential confounder. Finally, we performed a subgroup analysis using national health screening data from those who had undergone the relevant screening and further adjusted the results for objectively measured bio-clinical variables. In subgroup analysis, our results confirmed that adding DPP-4i to MET reduced the incidence of HHF compared to that on using MET+SU. In our population, the proportion of people with chronic kidney disease and diabetic micro-vascular complications (retinopathy, neuropathy, and nephropathy) was relatively low, suggesting that these results could reflect the risk of HHF in the early stage of T2D.

This study has several limitations. First, HHF was defined by an ICD-10 code from the NHIS health care utilization data, not by clinical or laboratory tests. Thus, there is the possibility of outcome misclassification. Second, the study had a relatively short follow-up duration. Further studies are needed to compare the long-term effects of second-line glucose-lowering drugs. Third, we did not examine safety profiles, such as diabetic ketoacidosis events or hypoglycemic events, during the follow-up period. Fourth, we did not compare the effects between the subclasses of DPP-4i. We also could not include new drugs such as sodium-glucose co-transporter-2 inhibitor, which was introduced in 2015, and glucagon-like peptide-1 receptor agonist, which was not covered by health insurance in Korea during the study period. Comparative studies of new emerging drugs that have recently started to be prescribed are expected in the near future. Fifth, in contrast to the SAVOR-TIMI 53 trial, in which DPP-4i was associated with higher risks of HHF, the protective effect of DPP-4i on HHF detected in this study could have resulted from the fact that we used SU as a reference for comparisons. Finally, we did not check glycated hemoglobin (HbA1c), reflecting the level of blood sugar controlled over the past 2 to 3 months, so we only adjusted for fasting glucose level.

Despite these limitations, the current study has several important strengths. The claims data related to ICD-10 codes and health screening data, which can reflect the biochemical and anthropometric status of patients, were combined to analyze the differences in the risks of HHF on using DPP-4i, TZD, and SU as add-on therapies to MET. We confirmed that DPP-4i added to MET reduced the risk of HHF despite the limitation of defining HHF by an ICD-10 code from NHIS claims data in the subgroup analysis. Smoking status and personal and family histories of CVD, which were determined via surveys during health screening, were also considered when analyzing the risk of HHF. This study was performed in a population of patients who started taking MET at diagnosis, which more closely reflects the common pattern of administration of oral glucose-lowering drugs under insurance coverage for the relatively early stages of T2D in Korea. It is worth analyzing the effect of add-on therapy patterns to MET using real-world data. Finally, we analyzed the risk of HHF people based on an underlying history of CVD.

## Conclusions

An add-on therapy to MET is often required for people with T2D who fail to respond to MET monotherapy. When trying to achieve glycemic control targets, HHF, a risk associated with CVD, should be considered when choosing from the available second-line drugs. This study of nationwide real-world data comparing add-on glucose-lowering medications suggests that DPP-4i added to MET might lower the risks of HHF compared to SU, irrespective of the patient's history of CVD. More investigative studies with longer follow-up durations assessing the drugs used in the present study or new and emerging drugs such as SGLT-2i or GLP-1 agonist are needed to better reflect real-world practice.

## Supporting information

S1 Table Hazard ratios for the development of heart failure by type of second-line antidiabetic medication in on-treatment analysis. (DOC)

S2 Table Baseline characteristics of second-line drugs in the subgroup with available health screening data. Data are reported as means ± standard deviations, medians [25th–75th percentiles], or numbers (percentages) unless otherwise stated. CVD, cardiovascular disease; MET, metformin; SU, sulfonylurea; DPP-4i, dipeptidyl peptidase-4 inhibitor; TZD, thiazolidinedione; HDL, high-density lipoprotein; LDL, low-density lipoprotein.
(DOC)

S1 Fig Cumulative risk of hospitalization of heart failure (HHF) events in Korean adults with type-2 diabetes from the Korean National Health Insurance database. (TIF)
