# Supplementary material for: Second-line glucose-lowering drugs added to metformin and the risk of hospitalization for heart failure: A nationwide cohort study
Source: PLoS One. 2019 Feb 11;14(2):e0211959. doi: 10.1371/journal.pone.0211959 (PMC6370220; doi:10.1371/journal.pone.0211959)
Supplement: S1 Table — (DOC) [file pone.0211959.s001.doc]

| **S1 Table. Hazard ratios for the development of heart failure by type of second-line  antidiabetic medication in on-treatment analysis** | | | | | | |
| --- | --- | --- | --- | --- | --- | --- |
| Drugs | Person-years | No. of cases | Event rate  (per 100,000 PY) | Adjusted 1* |  | Adjusted 2† |
|  |  |  |  | HR (95% CI) |  | HR (95% CI) |
| MET+SU | 71,041 | 341 | 480 | 1.00 |  | 1.00 |
| MET+DPP-4i | 79,588 | 279 | 351 | 0.84 (0.72 – 0.99) |  | 0.76 (0.64 – 0.90) |
| MET+TZD | 8,100 | 39 | 481 | 1.12 (0.80 – 1.55) |  | 1.09 (0.78 – 1.52) |
| MET, metformin; SU, sulfonylurea; DPP-4i, dipeptidyl peptidase-4 inhibitor; TZD, thiazolidinedione; HR, hazard ratio; PY, person-year; CI, confidence interval; no., number. *Adjusted for sex and age. †Adjusted for sex, age, the duration of metformin therapy, hypertension, dyslipidemia, atrial fibrillation, chronic kidney disease, microvascular complications of diabetes (retinopathy, neuropathy, or nephropathy), cardiovascular disease, the Charlson Comorbidity Index, and calendar index year. | | | | | | |
