# Supplementary material for: Second-line glucose-lowering drugs added to metformin and the risk of hospitalization for heart failure: A nationwide cohort study
Source: PLoS One. 2019 Feb 11;14(2):e0211959. doi: 10.1371/journal.pone.0211959 (PMC6370220; doi:10.1371/journal.pone.0211959)
Supplement: S2 Table — Data are reported as means ± standard deviations, medians [25th–75th percentiles], or numbers (percentages) unless otherwise stated. CVD, cardiovascular disease; MET, metformin; SU, sulfonylurea; DPP-4i, dipeptidyl peptidase-4 inhibitor; TZD, thiazolidinedione; HDL, high-density lipoprotein; LDL, low-density lipoprotein. (DOC) [file pone.0211959.s002.doc]

**S2 Table. Baseline characteristics of second-line drugs in the subgroup with available health screening data**

| Variables | MET+SU (n = 14,444) | MET+DPP-4i (n = 18,912) | MET+TZD (n = 1,941) |
| --- | --- | --- | --- |
| Men | 7,982 (55.3) | 10,573 (55.9) | 1,147 (59.1) |
| Age, years | 58.4 ± 10.4 | 56.2 ± 10.0 | 56.0 ± 10.0 |
| Inclusion year |  |  |  |
| 2009 | 7,000 (48.5) | 9,304 (49.2) | 1,045 (53.8) |
| 2010 | 3,994 (27.7) | 5,483 (29.0) | 535 (27.6) |
| 2011 | 2,024 (14.0) | 2,608 (13.8) | 217 (11.2) |
| 2012 | 1,426 (9.9) | 1,517 (8.0) | 144 (7.4) |
| Body mass index, kg/m^2^ | 25.4 ± 3.3 | 25.3 ± 3.2 | 25.2 ± 3.3 |
| Waist circumference, cm | 86.0 ± 8.38 | 85.4 ± 8.3 | 85.4 ± 8.6 |
| Systolic blood pressure, mmHg | 129.2 ± 15.3 | 127.3 ± 14.8 | 127.6 ± 15.0 |
| Total cholesterol, mg/dL | 189.7 ± 39.8 | 182.7 ± 38.3 | 183.1 ± 38.2 |
| Triglyceride, mg/dL | 143.0 [102.0-206.0] | 135.0 [96.0-195.0] | 135.0 [96.0-195.0] |
| HDL cholesterol, mg/dL | 51.2 ± 23.0 | 51.0 ± 21.0 | 51.3 ± 20.7 |
| LDL cholesterol, mg/dL | 107.1 ± 50.9 | 101.7 ± 44.9 | 101.6 ± 40.8 |
| Fasting glucose, mg/dL | 133.0 [116.0-154.0] | 128.0 [114.0-146.0] | 126.0 [112.0-144.0] |
| Creatinine, mg/dL | 1.02 ± 1.03 | 0.98 ± 0.88 | 1.02 ± 0.97 |
| **Smoking status** |  |  |  |
| Never | 8,553 (59.2) | 11,084 (58.6) | 1,078 (55.5) |
| Former | 2,975 (20.6) | 4,369 (23.1) | 447 (23.0) |
| Current | 2,916 (20.2) | 3,459 (18.3) | 416 (21.4) |
| **Family history** |  |  |  |
| Stroke | 1,401 (9.7) | 2,100 (11.1) | 225 (11.6) |
| Heart disease | 607 (4.2) | 1,029 (5.4) | 89 (4.6) |
| History of CVD | 2,089 (14.5) | 2,622 (13.9) | 275 (14.2) |
| Charlson score, unit | 2.8 ± 1.6 | 2.8 ± 1.6 | 2.8 ± 1.5 |
| Data are reported as means ± standard deviations, medians [25^th^–75^th^ percentiles], or numbers (percentages) unless otherwise stated. | | | |
| CVD, cardiovascular disease; MET, metformin; SU, sulfonylurea; DPP-4i, dipeptidyl peptidase-4 inhibitor; TZD, thiazolidinedione; HDL, high-density lipoprotein; LDL, low-density lipoprotein | | | |
