# Supplementary figures and images for: Second-line glucose-lowering drugs added to metformin and the risk of hospitalization for heart failure: A nationwide cohort study
Source: PLoS One. 2019 Feb 11;14(2):e0211959. doi: 10.1371/journal.pone.0211959 (PMC6370220; doi:10.1371/journal.pone.0211959)

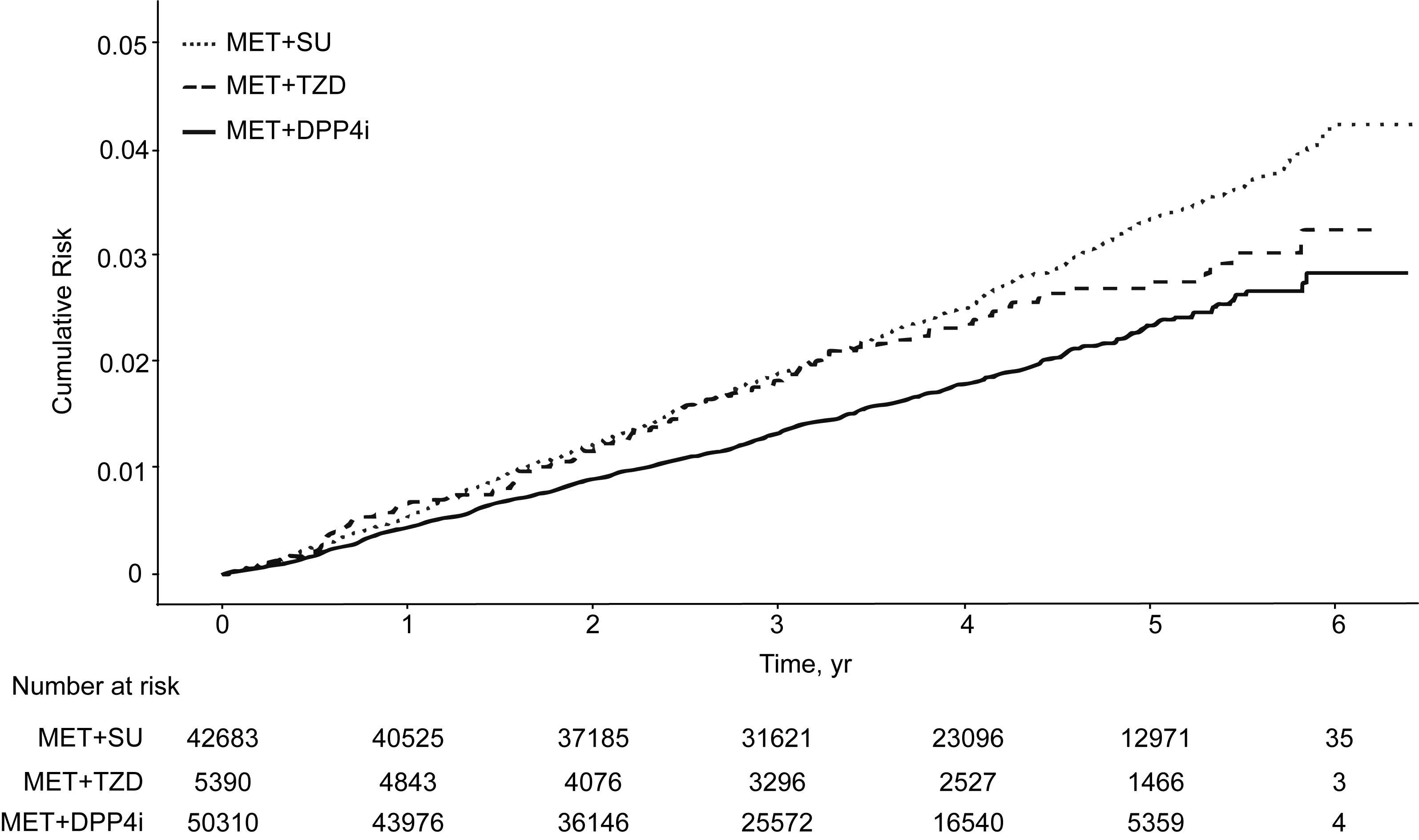

Supplement: S1 Fig — (TIF) [file pone.0211959.s003.tif]
